# Evaluation of the Toxicity Effects of Silk Fibroin on Isolated Fibroblast and Huvec Cells

**Published:** 2018

**Authors:** Parvaneh Naserzadeh, Seyed Alireza Mortazavi, Khadijeh Ashtari, Enayatollah Seydi, Jalal Pourahmad

**Affiliations:** a *Pharmaceutical Sciences Research Center, Shahid Beheshti University of Medical Sciences, Tehran, Iran.*; b *Department of Pharmaceutics, School of Pharmacy, Shahid Beheshti University of Medical Sciences, Tehran, Iran.*; c *Department of Medical Nanotechnology, Faculty of Advanced Technology in Medicine, Iran University of Medical Sciences, Tehran, Iran. *; d *Department of Occupational Health Engineering, Alborz University of Medical Sciences, Karaj, Iran.*; e *Department of Pharmacology and Toxicology, School of Pharmacy, Shahid Beheshti University of Medical Sciences, Tehran, Iran.*

**Keywords:** Silk Nanoparticles, Fibroblast, Huvec cells, Oxidative Stress, Mitochondria, Lysosomes

## Abstract

Emerging line research showed that silk nanoparticles (NPs) have toxicity on the fibroblast and Huvec cells without any toxicity recognized mechanisms. Recently, it suggested peripheral arterial disease confounds almost eight million Americans. Also, due to the main effect of fibroblast in a production of extracellular matrix (ECM), adhesive molecules, glycoproteins and various cytokines, it decided to define the toxicity mechanistic of silk NPs in fibroblast and Huvec cells based on oxidative stress markers. Therefore, it investigated whether silk NPs is able to induce any abnormality in the fibroblast and Huvec cells based on reliable and documented oxidative stress methods. Our results indicated that silk NPs (0.5, 1 and 2 mg/mL) induces cellular and mitochondrial dysfunction including an increase in ROS production, lipid peroxidation, mitochondria membrane potential (MMP) collapse, and oxidation of thiol groups which caused to cytochrome c release. Besides, lysosomal integrity damage and decreased in ATP/ADP ratio proposed disruptive effect of silk NPs on the mitochondrial respiratory chain and cell death signaling induction.

## Introduction

Silk fibroin is natural filament protein structure which is created by a diversity of insects and spiders with high strength, toughness, and elasticity power (1-3). Based on literature review, three-dimensional silk fibroin nets support the attachment, spread, and growth of a variety of human epithelial, fibroblast, keratinocyte and osteoblast of diverse tissue origins. In this context, the evidence indicates that the silks are used in drug delivery systems, bioengineering, and therapeutic delivery to cancer cells (4-7). The unique properties of silks fibroin such as excellent biocompatibility, controlled degradability, structural integrity, and versatile process ability offer it a suitable and useful candidacy to be incorporated in tissue engineering and drug delivery systems (4). Focusing on the peripheral arterial disease debilitated higher than eight million Americans and in recent years, silk fibroin is used as small caliber successor of the blood vessel (8). Pharmacological application of silk fibroin (SFNPs) has been reported in diabet, hypocholesterolemia, obesity, and wound healing therapy (4). Besides, numerous studies have been used in preclinical studies such as anti-oxidant and immune-regulatory, hair protecting action, anti-hangover, and anti-tumor effect (4). Recently, the toxicity mechanisms of SFNPs have been studied intensively due to inducible cell membrane and lysosomal damage characterization (4) Based on literature review, NPs induced oxidative stress affects cell signaling pathway: increasing in transcription of defense genes through nrf2, activation of inflammatory signaling through NF-ΚB, and activation of cell death pathways (9, 10). These NPs-induced ROS formation results in toxic altrations in a cell nucleus, DNA material,and mitochondria as major cell compartments (11-13). Mitochondria are sensitive organelles which are well known as the main source of ROS production in cells. NPs could enter to mitochondria and induce impairing structure and function of mitochondria which leads to excess ROS formation and oxidative stress induction (14). Taking into consideration the possible toxicity mechanisms of silk NPs, we planned to study the role of oxidative stress in silk fibroin NPs toxicity in huvec cells and fibroblast by carrying out different multi-parametric assays.

## Experimental

*Chemicals*

All chemicals were purchased from Sigma-Aldrich (St. Louis, MO) in the best pharmaceutical grade.

*Nanoparticle synthesis*

Silk fibroin was synthesized by Dr Mehdi Farokhi in National Cell Bank of Iran, Pasteur Institute of Iran, Tehran, Iran.

*Cell Culture*

Huvec cells (Life Technologies for Type Culture Collection, Wuhan, China) were cultured in RPMI 1640 medium (HyClone, UT, USA) containing 11.1 mmol/L glucose, 50 μmol/L β-mercaptoethanol, 1.0 mmol/L sodium pyruvate, 2.0 mmol/L L-glutamine, 100 U/mL penicillin, 100 μg/mL streptomycin (Sigma-Aldrich, MO, USA) and 10% fetal bovine serum (Gibco, MD, USA) in a humidified atmosphere of 95% air and 5% CO2 at 37 °C. Culture medium was replaced every 2 to 3 days. Upon reaching near confluence, cells were passaged with trypsin (0.25%) at a ratio of 1:3. Huvec cells used in this study were between passages 5-15.

*Cell viability assay*

Cell viability was assessed by 3-(4,5-dimethylthiazol-2-yl)-2,5- Diphenyl tetra zoliumbromide solution in RPMI-1640- medium and incubated for 6,12 and 18 h at 37 °C. At the end of the incubation period the plate was centrifuged (5 min, 800×g), and the untransformed MTT removed by carefully inverting, flicking and blotting the tray. Finally, DMSO, 100 µL, was added to each well. The absorbance was measured at 570 nm on ELISA reader (In finite 200 M, TECAN). Each concentration was tested in three different experiments run in three replicates for each sample (15).

*Isolation mitochondria of cell culture*

The cells were harvested in the late logarithmic phase of growth with a rubber policeman and centrifuged at 500×g for 5 min. The cell pellet was washed three times with 10 mL of serum-free Ham′s F-10 medium by repeated dispersion and centrifugation. The cell pellet was homogenized in 10 volumes of STM buffer (0.25 M sucrose; 20 mM Trist HCl; 1.1 mM MgCl_2_, at pH 7.85) by 15 strokes at 5000 rpm with a motorized pestle. The homogenate was centrifuged at 800×g for 10 min, and the resultant supernatant was then centrifuged at 16,000×g to obtain crude «mitochondrial» and «cytosol» fractions.

*Measurement of ROS*

The cells (1 × 10^6^) were treated with the appropriate dose of the silk NPs (0.5, 1 and 2 mg/mL) and with 10 mM of DCFH-DA. Intracellular reactive oxygen species (ROS) in the fibroblast and Huvec cells were determined after 6, 12, and 24 h of incubation at 37 °C by silk fibroin nanoparticles. The samples were centrifuged at 3000×g for 5 min to wash off the excess of silk nanoparticle. Then, the pellet was dissolved in 0.5 mL PBS, and also 100 µL aliquots were redistributed to the BD flowcytometry tube. The cells were read on the fluorescence and light scattering was analyzed for at least 10000 counts per sample in the flowcytometry using a BD Biosciences FACS Calibur TM flowcytometer. The samples were gated on the forward/side scatter to exclude cell debris and clumps. A flow cytometer with the Flowing software-2-5-1, equipped with a 488 nm argon ion laser, was used and fluorescence signals were obtained using a 530 nm bandpass filter (FL-1 channel) (16).

*Measurement of MMP*

Rhodamin123 (Rh 13) was used to measure the mitochondrial inner membrane potential (MMP). The cells were incubated with silk NPs (0.5, 1 and 2 mg/mL) for 6, 12, and 18 h. The samples were centrifuged at 3000×g for 5 min to wash off the excess of silk nanoparticle. The pellet was dissolved in 0.5 mL PBS, and 100 µL aliquots were redistributed to the BD flowcytometry tube. The cells were read on the fluorescence and light scattering was analyzed for at least 10000 counts per sample in the flowcytometry using a BD Biosciences FACS Calibur TM flow cytometer. The samples were gated on the forward/side scatter to exclude cell debris and clumps. A flow cytometer with the Flowing software-2-5-1, equipped with a 488 nm argon ion laser, was used and fluorescence signals were obtained using a 530 nm bandpass filter (FL-1 channel) (17). 

*Measurement of Lipid Peroxidation*

The cells (1 × 10^6^) were pre-incubated with an appropriate dose of the silk NPs (0.5, 1 and 2 mg/mL). In fibroblast and huvec cells, the lipid peroxidation was measured at 6, 12, and 24 after exposure to all mentioned concentration of silk NPs. Lipid peroxidation was initiated by addition of hydroxyl radical generating mixture. After an additional 30 min at room temp, 10% w/v of ice- cold TCA was added, and the samples were centrifugation 5 min at 3,000×g. The supernatant was collected (0.5 mL) and treated with TBA reagent (20 mM TBA in 50% v/v glacial acetic acid). The samples were then heated at 100 °C for 1 h. After the cooling period, butanol was added, and the organic layer was removed and redistributed to 96-well plates. The supernatant was evaluated at 532 nm with an ELIZA reader (Tecan, Rainbow Thermo, Austria) (16).

*Measurement of GSH and GSSG*

The reduced glutathione disulfide (GSH) to oxidized glutathione (GSSG) is a sensitive indicator of oxidative stress in the cells. Therefore, intracellular GSH and GSSG contents were determined based on a spectrofluorometric method by employing O-Phthalaldehyde (OPA) and N-Ethylmaleimide (NEM) probe. Aliquots of the cell suspension (0.5 mL) that were previously stained with OPA and NEM probe (5 µM) were separated from the incubation medium by centrifugation at 1000 rpm for 1 min. The cell pellet was then suspended in 2 mL of the fresh incubation medium. This washing process was carried out twice to remove the fluorescent dye from the media. Each sample was measured in quartz cuvettes using a Shimadzu RF5000U fluorescence spectrophotometer set for at 495 nm excitation and 530 nm emission wavelengths (18).

*Measurement of Lysosomal membrane integrity assay*

The lysosomal membrane stability was determined from the redistribution of acridine orange as a fluorescent dye. Aliquots of the cell suspension (0.5 mL) that were previously stained with acridine orange (5 µM) were separated from the incubation medium by centrifugation at 1000 rpm for 1 min. The cell pellet was then suspended in 2 mL of the fresh incubation medium. This washing process was carried out twice to remove the fluorescent dye from the media. Finally, the acridine orange redistribution in the cell suspension was measured by the spectrophotometer set at 495 nm excitation and 530 nm emission wavelengths (19).

*Measurement of mitochondrial ATP and ADP*

The concentration of adenosine triphosphate (ATP) in mitochondria was determined by a bioluminescent somatic cell assay kit (sigma Aldrich. MO 63103, USA) at 12 h. The bioluminescence intensity was measured by a Sirius tube luminometer (Berthold Detection System, Germany).

*Measurement of Cytochrome c release*

The concentration of cytochrome c was determined through using the Quantikine Cytochrome c Immunoassay kit provided by R and D Systems, Inc. (Minneapolis, Minn.). Briefly, a monoclonal antibody specific for rat/mouse cytochrome c was pre-coated onto the microplate. Seventy-five microliter of the conjugate and 50 μL of standard and positive control were added to each well of the microplate. One microgram of protein from each supernatant fraction was added to the sample wells. 

All of the standards, controls, and samples were added to two wells of the microplate. After 2 h of incubation, the substrate solution (100 μL) was added to each well and incubated for 30 min. Then, 100 μL of the stop solution was added to each well; the optical density of each well was determined by the aforementioned microplate spectrophotometer set to 450 nm (ELISA reader, In finite 200 M, 

TECAN).

*Measurement of mitochondrial Cytochrome c oxidase activity*

In this study, the mitochondrial cytochrome c oxidase activity and mitochondrial outer membrane (MOM) were measured using a cytochrome-c oxidase assay kit (Sigma, St. Louis, MO). The experimental procedures were performed according to the manufacturer′s protocol.

*Statically Analysis*

Data were analyzed using one-way and two-way ANOVA tests followed by the post-hoc Tukey and Bonferroni tests, respectively. The results were presented as mean ± SD. of the triplicate samples. The minimal level of significance chosen was *P *< 0.05.

## Results

*Silk fibroin NPs reduced of cell viability*

As shown in Table 1, silk fibroin NPs (0.5-2 mg/mL) showed the significant decrease in cell viability in the fibroblast and Huvec cells compared to control group (*P *< 0.05). Also, our results supposed that fibroblast is more susceptible to silk NPs exposure than huvec cells.

*Silk fibroin NPs induced ROS production*

**Figure 1:**
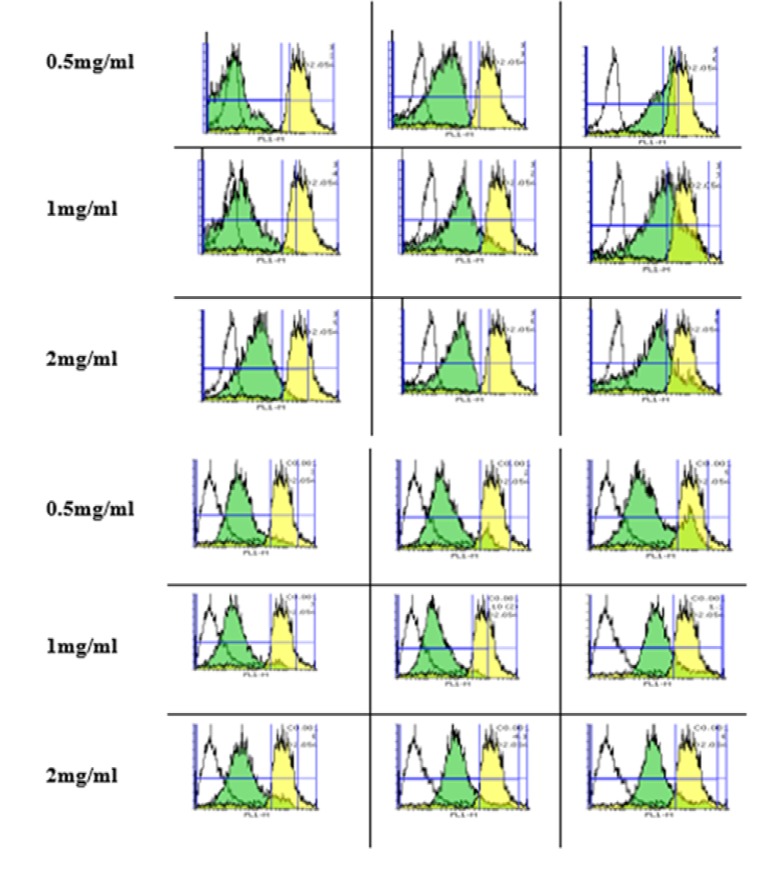
Silk fibroin induced ROS formation in isolated fibroblast and Huvec cells. ROS formation was determined by flow cytometry using DCFH-DA as described in materials and methods and demonstrated as fluorescence intensity of DCF. Values represented as mean

As shown in Figure 1, the rate of ROS formation significantly increased in a time and concentration-dependent manner after exposure to silk fibroin NPs compared to control mitochondria. Similar to the results of cell viability, fibroblast (Top) is more susceptible to silk NPs exposure than Huvec (Bottom) cells via shifting of DCF peak to the right. Besides, H_2_O_2_ (20 mM) was also used as positive control.

**Table 1:** The effect of silk fibroin on the cell viability:Cell viability was measured using MTT assay in fibroblast and Huvec cells (1 × 106 cells) were incubated for 6, 12 and 18 h with various concentrations of silk fibroin (0.5, 1 and 2 mg/mL).

**Groups**		**Cell viability (%)**		
		**6 h**	**12 h**	**18 h**
Fibroblast cells	Control	99.55 ± 0.44	99.47 ± 0.52	99.45 ± 0.55
	0.5 mg/mL	67.08 ± 2.54**	67.185 ± 4.85**	51.36 ± 4.78***
	1 mg/mL	63.21 **± **1.72**	63.51 **± **3.67**	46.625 **± **3.09***
	2 mg/mL	53.28 **± **2.20***	47.37 **± **5.25***	44 **± **1.76***
Huvec cells	control	99 **± **1	95.7 **± **5	95.7 **± **5
	0.5 mg/mL	67.08 ± 2.54**	68.05 ± 3.05**	41.2 ± 3.2***
	1 mg/mL	52.13 ± 2.13**	57.72 ± 2.72**	38.07 ± 3.07***
	2 mg/mL	51.45 ± 1.45**	47.37 ± 4.08***	29.90 ± 1.905***

Data represented as mean ± SD (n = 3).

** *P *< 0.01 and

*** *P *< 0.001 compared with control group.

**Table 2:** Effect of silk fibroin on lipid peroxidation in isolated fibroblast and Huvec cells. Fibroblast and Huvec cells (1 × 106 cells) were incubated for 6, 12 and 18 h with various concentrations of silk fibroin (0.5, 1 and 2 mg/mL). Data represented as mean ± SD of data determined from three separate experiments

**Groups**		**MDA (µg/mg protein)**		
		**6 h**	**12 h**	**18 h**
Fibroblast	control	7.327 ± 0.0045	7.3495 ± 0.0045	7.72 ± 0.017
	0.5 mg/mL	7.785 ± 0.003ns	7.751 ± 0.0045ns	7.659 ± 0.003ns
	1 mg/mL	7.828 ± 0.007*	7.992 ± 0.004*	8.048 ± 0.0025*
	2 mg/mL	8.246 ± 0.0015**	8.258 ± 0.0025**	8.252 ± 0.004**
Huvec	control	6.88 ± 0.027	7.091 ± 0.003	7.133 ± 0.001
	0.5 mg/mL	7.449 ± 0.004*	7.627 ± 0.005*	7.802 ± 0.003*
	1 mg/mL	7.949 ± 0.015*	7.866 ± 0.0115*	7.898 ± 0.008*
	2 mg/mL	8.111 ± 0.023**	8.159 ± 0.003**	8.160 ± 0.005**

Values represented as mean ± SD (n = 3).

* *P *< 0.05 and

** *P *< 0.01 compared with control group.

**Table 3:** Effect silk fibroin on mitochondrial GSH and GSSG content. The isolated fibroblast and Huvec cells (1 × 106 cells) were incubated for 6, 12 and 18 h with various concentrations of silk fibroin (0.5, 1 and 2 mg/mL). Data represented as mean ± SD of data determined from three separate experiments

**Groups**		**GSSG/GSH ratio**		
		**6 h**	**12 h**	**18 h**
Fibroblast	control	15.48 ± 0.097	15.48 ± 0.096	15.47 ± 0.097
	0.5 mg/mL	25.15 ± 0.092*	35.35 ± 0.10**	35.25 ± 0.124**
	1 mg/mL	28.19 ± 0.11*	35.09 ± 0.15**	41.06 ± 0.150**
	2 mg/mL	45.112 ± 0.17***	48.036 ± 0.021***	54.97 ± 0.204***
Huvec	control	25.74 ± 0.096	25.76 ± 0.098	25.73 ± 0.098
	0.5 mg/mL	34.77 ± 0.117**	35.17 ± 0.114**	35.51 ± 0.101**
	1 mg/mL	34.99 ± 0.120**	38.54 ± 0.133**	44.33 ± 0.133***
	2 mg/mL	39.60 ± 0.161***	40.17 ± 0.169***	46.616 ± 0.187***

Values represented as mean ± SD (n = 3).

* *P *< 0.05,

** *P *< 0.01 and

*** *P *< 0.001 compared with control group.

**Table 4:** Effect silk fibroin on cellular ADP/ATP level fibroblast and Huvec cells. Cells (1 × 106 cells) were incubated with silk fibroin (0.5, 1 and 2 mg/mL) and ADP/ATP level was determined using Luciferin/Luciferase Enzyme System as described in Materials and methods. Data represented as mean ± SD of data determined from three separate experiments

**Groups**		**ADP/ATP ratio**
		**12 h**
Fibroblast	control	0.1
	0.5 mg/mL	3.2 ± 0.4*
	1 mg/mL	42 ± 1.17*
	2 mg/mL	75 ± 1.2***
Huvec	control	1.17 ± 1
	0.5 mg/mL	12.6 ± 1*
	1 mg/mL	20.3 ± l l2**
	2 mg/mL	41.8 ± 1**

Values represented as mean ± SD (n = 3).

* *P *< 0.05,

** *P *< 0.01 and

*** *P *< 0.001 compared with control group.

**Table 5:** Effect of silk fibroin on mitochondrial outer membrane fibroblast and Huvec cells. The fibroblast and Huvec cells (1 × 106 cells) were incubated for 6, 12 and 18 h in the presence of different concentrations of silk fibroin and MOM integrity was measured as described in Materials and Methods

**Groups**		**Mitochondrial Outer Membrane damage**		
		**6 h**	**12 h**	**18 h**
Fibroblast	control	0.100 **± **0.005	0.101 **± **0.005	0.102 **± **0.01
	0.5 mg/mL	0.167 **± **0.0113*	0.206 **± **0.011**	0.2564 **± **0.006**
	1 mg/mL	0.226 **± **0.006**	0.285 **± **0.003**	0.306 **± **0.003***
	2 mg/mL	0.315 **± **0.003***	0.336 **± **0.006***	0.358 **± **0.008***
Huvec	control	0.32 **± **0.002	0.372 **± **0.005	0.404 **± **0.003
	0.5 mg/mL	0.374 **± **0.004*	0.405 **± **0.005**	0.466 **± **0.011**
	1 mg/mL	0.405 **± **0.004**	0.414 **± **0.004**	0.472 **± **0.006**
	2 mg/mL	0.420 **± **0.0025***	0.461 **± **0.005***	0.514 **± **0.003***

Values represented as mean ± SD (n = 3).

* *P *< 0.05,

** *P *< 0.01; and

*** *P *< 0.001 compared with control group.

**Table 6:** Effect of silk fibroin on cytochrome c release fibroblast and Huvec cells. The mitochondrial on fibroblast and Huvec cells (1 × 106 cells) were incubated for 12 h with various concentrations of silk fibroin. The cytochrome c release was measured using Cytochrome c ELISA kit as described in Materials

**Groups**		**cytochrome c release**
		**12 h**
Fibroblast	control	11.81 ± 0.002
	0.5 mg/mL	12.6 ± 0.003ns
	1 mg/mL	13.4 ± 0.002ns
	2 mg/mL	45.6 ± 0.002**
Huvec	control	13.13 ± 0.0005
	0.5 mg/mL	17.08 ± 0.004ns
	1 mg/mL	26.15 ± 0.002*
	2 mg/mL	47.70 ± 0.0125**

Values represented as mean ± SD (n = 3).

* *P *< 0.05 and

** *P *< 0.01 compared with control mitochondria.

± SD (n = 3). [White peak: Control; Green peak: test; Yellow peak: H2O2 (positive control)].

**Figure 2:**
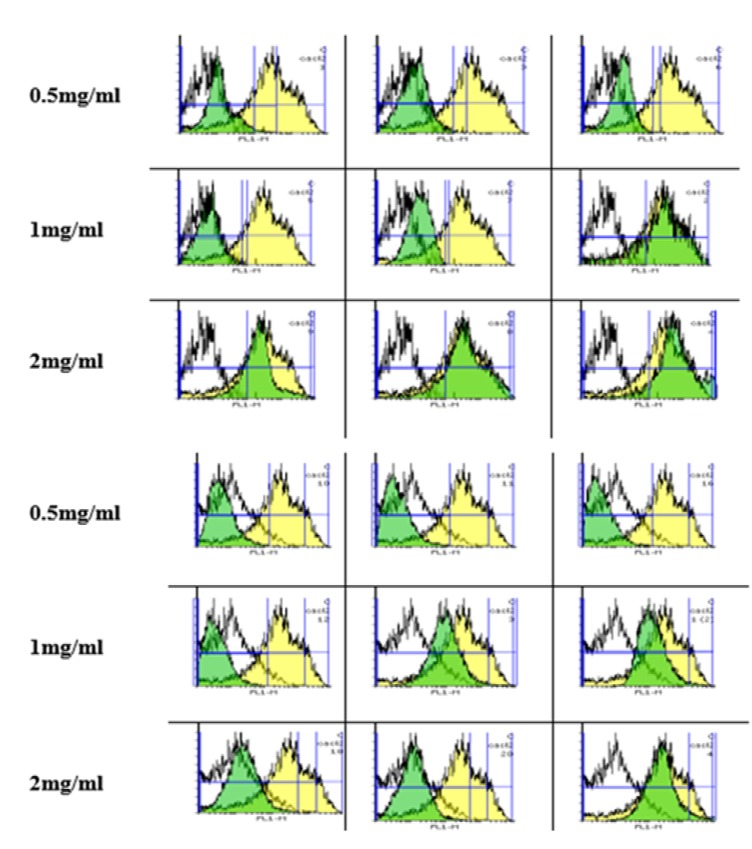
Effect of silk fibroin on mitochondrial membrane potential MMP collapse (ΔΨ%) in fibroblast and Huvec cells. Membrane potential collapse (ΔΨ%) was measured by Rhodamine 123 as described in Materials and Methods. The values are expressed as means

± SD (n = 3). [White peak: Control; Green peak: test; Yellow peak: H2O2 (positive control)].

**Figure 3:**
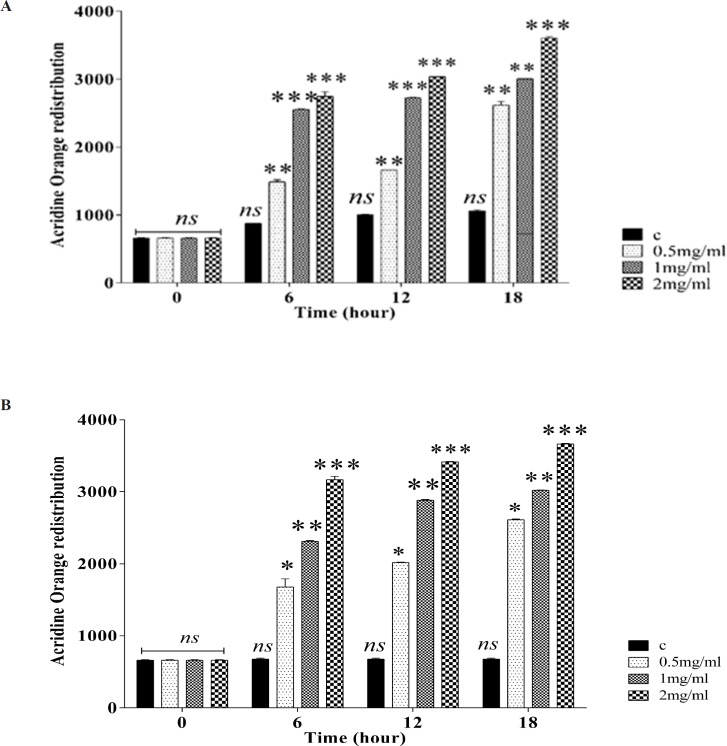
Effect of silk fibroin on lysosomal membrane damage. Lysosomal membrane damage was determined as the difference in redistribution of acridine orange from lysosomes into cytosol between the treated cells and control cells. Our data were shown as the percentage of lysosomal membrane leakiness in all the treated (test) fibroblast (A) and Huvec (B) cells. Values represented as mean ± SD (n = 3). *P < 0.05, **P < 0.01 and ***P < 0.001 significantly compared to corresponding control group

*Silk fibroin NPs declined MMP*

The uptake of Rh 123 has been used for the measurement of MMP collapse based on shifting of the Rh 123 peak to the right and an increase in AUC. 

As shown in Figure 2, there was a significant difference in the MMP collapse between the control and fibroblasts (Top) and Huvec) cells (Bottom groups after expossure to silk fibroin NPs (0.5-2 mg/mL; *P *< 0.05). Also, our results supposed that fibroblast is more susceptible to silk NPs exposure than Huvec cells in MMP collapse.

*Silk fibroin NPs induced lipid peroxidation*

As shown in Table 2, silk fibroin NPs (0.5-2 mg/mL) significantly (𝑃 < 0.05) induced MDA production (as marker of lipid peroxidation) in fibroblast and Huvec cells compared to corresponding control groups. Also, our results supposed that fibroblast is more susceptible to silk NPs exposure than Huvec cells in increasing of MDA level.

*Silk fibroin NPs changed cellular glutathione level*

Decreased in GSH and increase in GSSG level after incubation of fibroblast and Huvec cells with silk fibroin NPs (0.5-2 mg/mL) has been showed compared to the control group (Table 3). In agreement with other results, fibroblast was more susceptible to silk NPs exposure than Huvec cells on GSH/GSSG ratio.

*Silk fibroin NPs increased lysosome damage*

As shown in Figuresfibroblasts (A) and Huvec cells (B), lysosomal damage significantly increases after incubation of fibroblast and Huvec cells after incubation with silk fibroin NPs 

(0.5-2 mg/mL). Our results supposed that fibroblast is more susceptible to silk NPs exposure than Huvec cells in lysosomal damage.

*Silk fibroin NPs changed ATP and ADP levels*

Mitochondrial electron transfers chain (mETC) is required for ATP production. As shown in Table 4, silk fibroin NPs (0.5-2 mg/mL) significantly decreased the ATP level and ATP/ADP ratio from fibroblast and Huvec cells compared to the control group, indicating mitochondrial dysfunction (*P *< 0.05) (Table 4).

*Silk fibroin NPs impairing mitochondrial outer membrane (MOM) integrity*

As shown in Table 5, MOM was significantly decreased after the exposure to silk fibroin NPs (0.5-2 mg/mL) in a concentration-dependent manner compared to the control group (*P *< 0.05).

*Silk fibroin NPs increased cytochrome c release*

As shown in Table 6, silk fibroin NPs (0.5-2 mg/mL) induced significant expulsion of cytochrome c from mitochondria obtained from fibroblast and Huvec cell.

## Discussion

Several lines of evidence have demonstrated that the silks are naturally occurring protein polymers used for drug delivery, controlled drug release, medical imaging, biochemical sensors, tissue engineering, and regeneration in natural fiber form, while after processing *in-vitro* they can be assembled into several morphological forms (20, 21). Moreover, since currently the most usage of silk fibroin NPs is an issue in biomedical, it must be considered in evaluation f toxicity in organelles and sub-organelles in the body. It is widely accepted that oxidative stress has a key role in silk fibroin NPs toxicity in cells via ROS overproduction (21). Also, these observations raised the issue that mitochondria have the central role in ROS formation, we planned to study the role of oxidative stress in silk fibroin NPs in two different cell line (fibroblast and Huvec cells) by carrying out different multi-parametric assays. Therefore, in the present study, Huvec cells which are given from peripheral arterial tissue and also, fibroblasts as the main compartment of cells were considered as an optimal model *in-vitro* in the present study to assess the toxicity of silk fibroin NPs.

In the present study, we observed several important findings which confirm other inline previous studies: At the first step, MTT assay was used as a common method for assessing the cytotoxicity based on the mitochondrial complex II activity, which influences metabolic activity and cell viability in fibroblast and Huvec cells. Our data showed viability was decreased after exposure to silk fibroin NP 

(0.5-2 mg/mL) following 6, 12, and 18 h incubation (*P *< 0.05). These findings similar to basic documents suggest the reduction in mitochondrial complex II activity is related to amelioration in mitochondrial respiration rates which would be helpful in apprising of mitochondrial dysfunction (15). Other important mechanisms of NP-induced oxidative stress is related to ROS generation via the Fenton reaction which produced both in normal and pathological term (13, 22) as well as our findings revealed rapid rise in ROS formation in fibroblast and Huvec cells following exposure to silk fibroin NP (1 and 2 mg/mL) after 6, 12, and 18 incubation which certified involvement of ROS formation in Silk fibroin NP-induced mitochondrial dysfunction. Accumulating evidence *in-vivo* and *in-vitro* studies demonstrated that the increased ROS generation caused by lipid membrane attack, especially in mitochondria and cell membranes, leads to lipid peroxidation. Furthermore, we found that the silk fibroin NP (1 and 2 mg/mL) after 6, 12, and 18 incubation induces oxidation of the main products of lipid membrane, particularly malondialdehyde (MDA) formation (23). It was supposed that the oxidation of lipid membranes could result in disruption of electron transfer chain (ETC) and consequently increased ROS formation and oxidation of thiol groups in MPT pore and MMP event as mitochondrial dysfunction biomarker. Mitochondrial membrane permeability is controlled mainly by controlled proton movement across the inner mitochondrial membrane via regulation of voltage-dependent anion channel (VDAC) (24). In parallel, we also demonstrated that silk fibroin NPs 

(0.5-2 mg/mL) induced a marked decrease of MMP collapse event followed by outer membrane damage and uncoupling of oxidative phosphorylation in agreement with previous studies (23). It is well documented that GSH is a required component which protects mitochondria against MPT pore opening and oxidative stress induction (23). A survey of the literature indicates that GSH is required for maintenance of reduced form of thiol groups which protects against MPT (25). Moreover, in the section else of this study we have exhibited a significant GSH oxidation in a dose-dependent manner in agreement with previous investigations (23, 26). Mitochondria are often referred to as energy powerhouses for their critical roles in energy production in eukaryotic cells. Therefore, measurement of ATP level via oxidative phosphorylation process helps to determinate cell death mode in cells (27). Therefore, regarding to this subject, our outcome indicated that silk fibroin NPs (0.5-2 mg/mL) inhibited mitochondrial ATPase which decreased the ATP production and increased ADP level. 

The remaining ATP was immediately consumed for the maintenance of MMP, leading to further reduction of ATP concentration and mitochondrial dysfunction (15). This process has resulted in an increase in the LPO, decrease in the level of GSH decrease the MMP, and eventually decrease in ATP level. 

The decrease in MMP redounds in MPT opening pore and cytochrome c explosion from mitochondria to cytosol. Therefore, we further assayed the cytochrome c explosion after exposure with silk fibroin NPs. Also we observed that silk fibroin NPs (0.5-2 mg/mL) release of cytochrome c as key role in the apoptosis signaling in full agreement with previous studies (26, 27). In order to monitor the impact of silk fibroin NPs on the lysosomes damage, fibroblast and Huvec cell lysosomes were loaded with acridine orange (a lysosomotropic agent), a significant release of acridine orange into the cytosolic fraction ensued after incubation with silk fibroin NPs (0.5-2 mg/mL) indicating a severe damage to lysosomal membrane. Our data showed the re distribution of acridine orange from lysosomes into cytosole in both fibroblasts and Huvec cells, suggesting increased lysosomal membrane leakiness, consistent with the mitochondrial swelling and rise in ROS production (28). Therefore, we supposed that oxidative stress in silk fibroin is related to cross-talk reaction between mitochondria and lysosomes. 

The lysosomal proteases such as cathepsins have an extensive range of degradation roles within the lysosomes and are usually present in the cytosol in abnormality condition which leads to apoptotic, autophagic, and cell cycle arrest (19, 28 and 29). 

## Conclusion

In conclusion, this study indicated that silk Fibroin NPs impaired the electron transfer chain in fibroblast and Huvec cell line which leads to rising in ROS production lipid peroxidation, GSH depletion, and failure in oxidative phosphorylation. 

The possible mechanisms underlying the cell death signaling of Silk fibroin might be attributed to ATP cellular declination, loss of MOM integrity, MMP disruption and cytochrome c expulsion from mitochondria, which might be related to mitochondrial and lysosomal damage.
